# Quantitative Assessment of Intralesional Bleomycin for Periungual Warts in Nicaragua: A Retrospective Case Series

**DOI:** 10.7759/cureus.101224

**Published:** 2026-01-10

**Authors:** Virgilio Blandon, Maria-Jose Altamirano, Alessandro Alvarado, Taylys A Leyton, Inder Zelaya

**Affiliations:** 1 Dermatology, Centro Nacional de Dermatologia, Managua, NIC; 2 Dermatology, Universidad Nacional Autonoma de Nicaragua (UNAN), Managua, NIC; 3 Dermatology, Universidad Americana, Managua, NIC

**Keywords:** cryotherapy, human papillomavirus, intralesional bleomycin, periungual warts, retrospective case series

## Abstract

Background: Periungual warts, caused by human papillomavirus (HPV) infection, are notoriously difficult to treat due to high recurrence rates and resistance to conventional therapies, significantly impacting patient comfort and daily activities. Although intralesional bleomycin has shown promise for resistant warts, data from Central America are scarce. Furthermore, most existing efficacy assessments rely on subjective clinical scales or binary outcomes, with few studies employing serial, quantitative measurements of lesion area.

Objective: This study aimed to evaluate the therapeutic outcomes and safety profile of intralesional bleomycin in the management of periungual warts, using quantitative lesion area assessment (cm²) to objectively measure treatment response.

Methods: We conducted a retrospective case series of 15 patients diagnosed with periungual warts who received intralesional bleomycin therapy at the National Dermatology Center of Nicaragua between May 2020 and November 2022. Therapeutic response was assessed by percentage reduction in total lesion area (cm²) and number of lesions. Adverse events, treatment parameters, and duration were also recorded.

Results: Regarding efficacy, 60% (9/15) of patients achieved complete remission (100% reduction in lesion area), 33.3% (5/15) showed an 80%-99% reduction, and 6.7% (1/15) experienced a 50%-79% reduction. Most patients (66.7%, 10/15) received two therapeutic sessions, with a total treatment duration of eight weeks in 66.7% (10/15) and four weeks in 33.3% (5/15). Adverse events were reported in 86.7% (13/15) of patients, most commonly pain (80%, 12/15), erythema (40%, 6/15), and post-inflammatory hyperpigmentation (33.3%, 5/15). Less frequent events included onychomadesis (20%, 3/15), edema (6.7%, 1/15), pruritus (6.7%, 1/15), and permanent partial nail plate loss (6.7%, 1/15). Among patients who achieved complete remission, no recurrences were observed during the six-month follow-up. The quantitative lesion area measurement confirmed a statistically significant reduction in lesion size across the cohort.

Conclusion: Intralesional bleomycin appears highly effective for periungual warts, achieving substantial reductions in quantitatively measured lesion area and high complete remission rates, with a mostly transient adverse effect profile. This study is among the first in Central America to report on bleomycin treatment for periungual warts and is, to our knowledge, the first to evaluate efficacy using serial quantitative measurements of lesion area (cm²).

## Introduction

Periungual warts, caused by cutaneous human papillomavirus (HPV), are common and often highly recalcitrant lesions of the ungual unit [1,2]. While typically benign, certain HPV types (e.g., HPV 16, HPV 18) carry a rare but significant risk of malignant transformation to squamous cell carcinoma [1,3]. Their anatomical location, adjacent to the nail plate, poses an inherent therapeutic challenge; viral entry is habitually facilitated by minor trauma or maceration, and host immunity plays a critical role in their persistence [2-4]. This leads frequently to treatment resistance, increased recurrence rates, and significant aesthetic or functional morbidity [4,5]. Notwithstanding a wide array of therapeutic modalities globally, no single treatment has demonstrated universal efficacy or an ideal safety profile for these challenging lesions [2,4,6-8].

Intralesional bleomycin has emerged internationally as a promising alternative for recalcitrant warts, including periungual types [2,4,7,9]. A comprehensive 2020 systematic review, for instance, demonstrated significantly higher complete cure rates with intralesional bleomycin injections compared to saline or cryotherapy for common warts [9]. This robust evidence, coupled with reports of high clearance rates and potentially fewer treatment sessions for various recalcitrant wart types, including periungual lesions, solidifies bleomycin's potential role where conventional treatments underperformed [10-12]. Through its direct cytotoxic effects, bleomycin offers a distinct advantage in targeting persistent viral lesions, making it a valuable option for cases unresponsive to standard care [13].

Nevertheless, real-world evidence regarding the efficacy and safety of intralesional bleomycin for periungual warts in specific populations, particularly in Nicaragua, is extremely limited. To our knowledge, this is the first case series reported in both Central America and the Caribbean. This knowledge gap impedes evidence-based therapeutic decisions and limits understanding of bleomycin's performance across diverse demographics.

Beyond this geographic underrepresentation, another important limitation of the existing literature is its predominant reliance on dichotomous or semiquantitative outcome measures, such as complete clearance versus persistence, reduction in lesion count [14-18], or categorical multilevel scales based on estimated percentage reduction (e.g., complete, partial, or no response) [19-27]. Although these approaches reflect clinical judgment, they lack the objectivity and sensitivity of direct, serial measurements. As a result, objective serial quantification of lesion area has not been reported in the literature on periungual wart treatment. This gap is particularly relevant for periungual warts, in which resolution is often gradual and clinically meaningful partial responses are common. In this context, quantitative assessment of changes in lesion area would allow a more sensitive, reproducible, and granular evaluation of treatment efficacy, capturing therapeutic improvements that binary clearance-based outcomes may fail to detect.

Addressing both gaps, this retrospective case series presents initial therapeutic outcomes in 15 patients with periungual warts treated with intralesional bleomycin at the National Dermatology Center in Nicaragua, incorporating objective measurement of lesion area (cm²) to assess treatment response. Our findings aim to contribute regional data from an underrepresented population while also introducing a more precise and quantitative approach to outcome assessment in the management of recalcitrant periungual warts.

## Materials and methods

Study design and setting

This manuscript reports a retrospective analysis of the bleomycin treatment arm from a thesis project originally designed as a randomized clinical trial comparing intralesional bleomycin versus cryotherapy for periungual warts. The original trial (30 patients, 15 per group) was conducted at the National Dermatology Center of Nicaragua between May 2020 and November 2022 [28]. All extracted clinical variables and bibliographic data were compiled into a structured database and managed using IBM SPSS Statistics for Windows, Version 27.0 (Released 2019; IBM Corp., Armonk, NY, USA) as part of the original thesis framework, ensuring standardized data entry, traceability, and reproducibility of analyses.

Several factors necessitated analyzing only the bleomycin cohort. First, there was significant and irrecoverable data loss in the cryotherapy group database; second, regulatory constraints, including the absence of a national clinical trial approval mechanism (Comité Nacional de Investigación en Salud (CONIS)) at the study's inception; and third, the original trial was not formally registered in a public database, reflecting the limited research infrastructure available in Nicaragua at that time. Therefore, this report constitutes a retrospective case series focusing on the well-documented bleomycin cohort.

Patient population

The study included 15 consecutive patients randomized to the bleomycin arm within the original trial. Selection was based on strict criteria, including a dermatoscopic diagnosis of periungual warts with an evolution exceeding three months, age between 18 and 65 years, and residence in Managua. Diagnosis was confirmed by the presence of verrucous papules or plaques in the periungual location, supported by dermatoscopic findings such as thrombosed capillaries (black dots) and a papillomatous surface; no biopsies were performed. Patients were excluded if they had received prior treatment in the preceding four weeks, presented with lesions larger than 10 cm^2^, were pregnant, were HIV-positive, or had any history of contraindicating conditions such as Raynaud's disease or hypersensitivity to bleomycin. All included participants provided informed consent and committed to receiving exclusive care at the National Dermatology Center.

Data collection

Data extraction encompassed all relevant baseline characteristics, including demographic variables (age, sex, and educational attainment) and comprehensive clinical data. Clinical variables included wart duration, lesion characteristics (initial and final number of lesions, and location on the affected hand/digit), and total affected area (initial and final, quantified in cm^2^ by measuring the longest diameter and perpendicular width using a flexible tape measure and applying an estimation formula). Treatment details (number of bleomycin sessions administered), treatment outcomes (remission, recurrence, and response), and any treatment-related adverse events were systematically recorded from the medical charts. All 15 patients completed the full treatment and follow-up protocol, resulting in no missing data for primary and secondary outcomes.

Treatment protocol

Bleomycin sulfate (15 units per vial) was initially diluted in 5 mL of isotonic saline. The reconstituted bleomycin (CELON LABS) was then reconstituted with 2% lidocaine without epinephrine at a 2:1 ratio (two-parts lidocaine to one-part reconstituted bleomycin), achieving a final injectable concentration of 1 U/mL. Using a 30-gauge needle, the solution was injected intralesionally via a single puncture at the base of each wart until blanching was achieved, with the following dosing constraints: a maximum of 1 mL per digit over the entire treatment course, a maximum of 1 mL administered per session, and a total cumulative dose not exceeding 2 mL per patient. The injections were performed to create a dermal depot at the base of the lesion. Small lesions received a single central injection at their base. For larger lesions, the dose was meticulously distributed in a quadrant pattern (four injections aligned to the cardinal points) to facilitate comprehensive infiltration.

Patients received a maximum of two treatment sessions, spaced four weeks apart. Treatment was discontinued upon achieving complete clinical remission or after the second session. All patients who achieved complete clinical resolution were followed for a period of six months to monitor for recurrence. Patients with residual lesions after two bleomycin sessions were offered alternative therapies until resolution (these subsequent treatments were outside the scope of the original trial).

Outcome measures

The primary outcome of this retrospective analysis was the rate of complete clinical remission (cure rate), defined as the total resolution of verrucous tissue and restoration of normal skin and nail anatomy. This was assessed by the treating dermatologist at a follow-up visit four weeks after the second treatment session. The secondary outcomes included the evaluation of the sustained cure rate during the six-month follow-up period to ascertain recurrence. We also assessed the overall response to treatment based on changes in lesion size (quantified in cm^2^) and number; the treatment's efficiency was documented by tracking the number of sessions required to achieve complete remission, and recorded the incidence of treatment-related adverse events from patient reports in the medical records.

Statistical analysis

Descriptive statistics were used to summarize the data. Categorical variables are presented as frequencies and percentages.

Continuous variables (age, wart duration, initial and final number of lesions, and initial and final total affected area) were first tested for normality using the Shapiro-Wilk test. Given the small sample size (n = 15) and the resulting non-normal distribution of continuous data, these variables are presented as the median and interquartile range (IQR).

To evaluate the efficacy of the therapy, we compared the changes in the primary measures of lesion burden: initial versus final number of lesions and initial versus final total affected area. Prior to comparison, the assumption of symmetry was evaluated by calculating the Z-score of skewness (skewness/standard error).This assessment determined the appropriate non-parametric test: the Wilcoxon signed-rank test was used for symmetric distributions, while the Sign test was employed for significantly asymmetric distributions (|Z-skewness| > 1.96) to maintain accurate Type I error control.

The non-parametric Wilcoxon signed-rank test (or the exact Sign test, based on the Z-score) was used for these paired comparisons. Due to the small sample size, the exact p-value was calculated for both tests, setting a time limit of five minutes for the computation. The effect size (r) for the Wilcoxon test was calculated and reported to contextualize the magnitude of the observed difference. A Bonferroni correction threshold of p < 0.025 was applied because the significance level (α = 0.05) was divided by the number of primary lesion burden outcomes being tested (n = 2: total affected area and number of lesions).

A two-tailed p-value < 0.05 defined statistical significance for all analyses, including the Shapiro-Wilk test for normality. All tests were performed using IBM SPSS Statistics for Windows, Version 27.0 (Released 2019; IBM Corp., Armonk, NY, USA).

Ethical considerations

This study involved a retrospective review of clinical records without direct patient intervention. In accordance with the international ethical standards for observational research, and given the minimal risk nature of the study, individual informed consent for the retrospective analysis was waived. The study was conducted with approval from the hospital administration of the National Dermatology Center of Nicaragua, following their established protocols for medical record-based research.

All patients had previously provided written informed consent for the intralesional bleomycin procedure itself, which included a detailed explanation of the treatment's risks, benefits, and alternatives.

While Nicaragua lacked a formal Institutional Review Board (IRB) structure at the time of the study, we adhered to the ethical principles of the Declaration of Helsinki regarding confidentiality and data protection. All direct and indirect patient identifiers were removed prior to data collection, and results are presented only in aggregate form to ensure patient privacy. This approach is consistent with established ethical practices for retrospective studies in settings with developing research infrastructure.

We acknowledge the lack of formal IRB approval as a limitation inherent to conducting research in this specific context and have taken all available measures to ensure participant protection and data security.

## Results

Baseline characteristics of the study cohort

A total of 15 participants were included in this study. The baseline demographic and clinical characteristics of the cohort are summarized (Table 1).

**Table 1:** Baseline demographic and clinical characteristics of the study participants (N = 15) This table summarizes the baseline characteristics of the cohort. Categorical variables are presented as frequencies (n) and percentages (%). Continuous variables, which were all non-normally distributed based on the Shapiro-Wilk test (statistical significance defined as p < 0.05), are reported as median and interquartile range (IQR).

Characteristic	Category	Frequency (n)	Percentage (%)
Sex	Female	9	60
	Male	6	40
Academic level	High school	6	40
	University	9	60
Lesion location	Right hand	10	66.7
	Left hand	1	6.7
	Both hands	4	26.7
Continuous variables	Median	IQR	p (Shapiro-Wilk)
Age (years)	24	20-32	0.001
Duration of wart (months)	10	7-15	<0.001
Initial affected area (cm²)	0.7	0.5-2.8	0.001
Initial number of lesions	2	1-4	0.003
Number of sessions administered	2	1-2	<0.001

The cohort comprised nine females (60.0%) and six males (40.0%). In terms of academic attainment, the majority of participants (60.0%, n = 9) were at the university level, while six participants (40.0%) were at the high school level. Regarding the location of the lesions, the right hand was the most frequently affected site (66.7%, n = 10), followed by both hands (26.7%, n = 4), and the left hand (6.7%, n = 1).

Continuous variables are presented as median and interquartile range (IQR) due to a non-normal distribution, as confirmed by the Shapiro-Wilk test (all p-values < 0.05). The median age of the participants was 24 years (IQR: 20-32). The median duration of the warts prior to intervention was 10 months (IQR: 7-15). The median initial affected area was 0.7 cm² (IQR: 0.5-2.8), and the median initial number of lesions was 2 (IQR: 1-4). Participants received a median of two treatment sessions (IQR: 1-2).

Treatment efficacy and safety profile

The primary and secondary outcomes assessing treatment efficacy, efficiency, and safety of the treatment are summarized (Table 2). The intervention demonstrated an important efficacy, with complete clinical remission, which was the primary outcome, achieved in 60.0% of the cohort (n = 9). The remaining patients (40.0%, n = 6) exhibited a partial response, and no cases of treatment failure were recorded.

**Table 2:** Treatment efficacy, efficiency, and safety outcomes This table summarizes the primary efficacy, long-term follow-up, treatment efficiency, and safety profile of the intervention. The analysis for "follow-up and efficiency" (sustained cure and sessions to remission) is based on the subset of patients who achieved complete clinical remission (n = 9). All percentages for "efficacy" and "safety" are calculated from the total cohort (N = 15). An asterisk (*) denotes a permanent adverse event involving loss of approximately 4 mm of the lateral nail plate.

Outcome/adverse event	Statistics (n/N)	Percentage (%)
Efficacy (clinical response at final follow-up)		
Complete clinical remission (primary outcome)	9/15	60
Partial response	6/15	40
Follow-up and efficiency		
Sustained cure (no recurrence at six months)	9/9	100
Recurrence at six-month follow-up	0/9	0
Remission achieved with one session	5/9	55.6
Remission achieved with two sessions	4/9	44.4
Safety (adverse events)		
Patients experiencing any adverse event	13/15	86.7
Specific adverse event		
Pain (24h post-injection)	12/15	80
Erythema (24h post-injection)	6/15	40
Edema (24h post-injection)	1/15	6.7
Onychomadesis	3/15	20
Hyperchromia (hyperpigmentation)	5/15	33.3
Partial nail plate loss*	1/15	6.7

**Figure 1:**
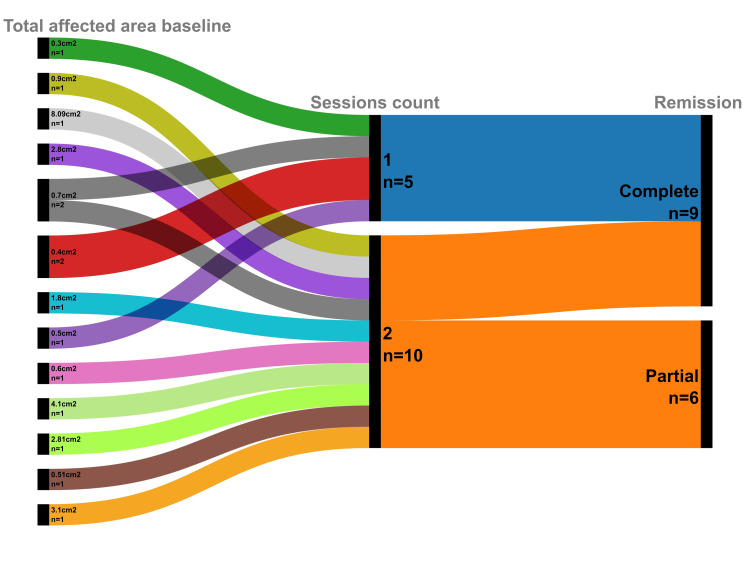
Patient pathways: baseline affected area, treatment sessions, and clinical outcomes This alluvial chart illustrates the flow of the 15 study participants from their baseline affected area (left), through the number of treatment sessions administered (center), to their final clinical outcome (right). The width of the streams corresponds to the number of patients in each pathway, highlighting that most patients with a smaller initial area achieved complete remission, often with a single session.

Of the patients who achieved initial complete remission, 100% (n = 9) maintained a sustained cure throughout the six-month follow-up period, with no recurrences observed, as illustrated in the patient pathway analysis (Figure 1). The treatment regimen demonstrated notable efficiency, with over half of the responders (55.6%, n = 5) attaining remission after a single session, while the remainder (44.4%, n = 4) required a second session to attain complete clearance.

**Figure 2:**
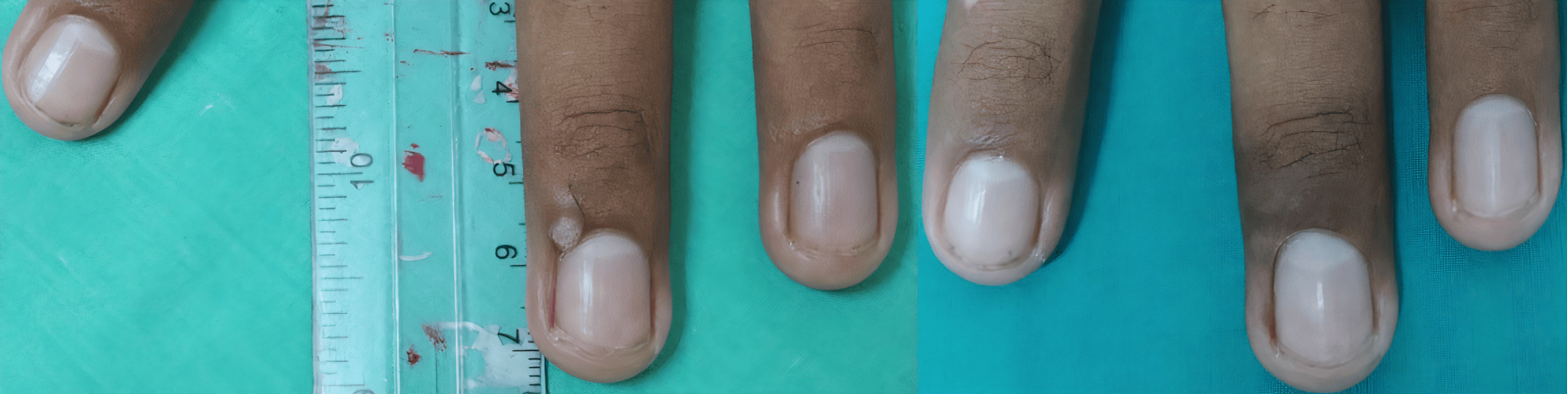
Transient post‑inflammatory hyperpigmentation after wart clearance The left image shows a periungual wart on the eponychium of the right third finger prior to treatment. The right image, taken two weeks after two sessions of intralesional bleomycin, demonstrates complete lesion resolution accompanied by marked hyperpigmentation at the treatment site. Clinical follow‑up confirmed that this hyperpigmentation subsequently resolved completely.

**Figure 3:**
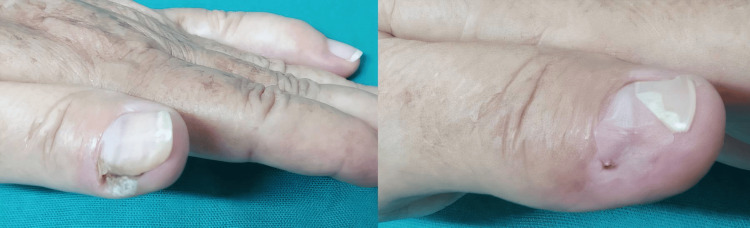
Complete clearance of a periungual wart with subsequent partial nail plate loss The left image shows the pretreatment appearance of a verrucous plaque on the lateral border of the left thumb. The right image, taken two weeks after two treatment sessions, demonstrates complete wart resolution accompanied by onychomadesis and partial nail plate loss. This partial nail plate loss (approximately 4 mm in length) remained unchanged and was evident throughout the six‑month follow‑up period.

The safety analysis revealed a characteristic profile of predominantly transient local reactions. The majority of patients (86.7%, n = 13) experienced at least one adverse event, the most prevalent being injection site pain within 24 hours post-procedure (80.0%, n = 12). Other frequently observed events included erythema (40.0%, n = 6) and hyperpigmentation (33.3%, n = 5) (Figure 2). Less common reactions included onychomadesis (20.0%, n = 3), and edema (6.7%, n = 1). One case of partial nail plate loss (6.7%, n = 1) resulted in permanent damage, involving approximately 4 mm of the lateral nail plate (Figure 3). All other adverse events were self-limiting and classified as mild to moderate in severity, resolving without medical intervention.

Objective measures of lesional burden

A quantitative analysis of lesional burden confirmed the notable reduction in disease after treatment (Table 3). Initial assessment confirmed non-normal distribution for all variables. To determine the appropriate statistical test for paired comparisons, we analyzed the symmetry of the differences between baseline and follow-up measurements using Z-skewness. Based on established thresholds (|Z| ≤ 1.96 indicating symmetry, |Z| > 1.96 indicating significant asymmetry), we selected the most appropriate test for each parameter.

**Table 3:** Change in objective measures of lesional burden from baseline to final follow-up This table presents the objective measures of disease burden before and after the intervention. Data are presented as median and interquartile range (IQR). The choice of statistical test for paired comparisons was determined by the symmetry of the differences between baseline and follow-up measurements, assessed using Z-skewness (where |Z| ≤ 1.96 indicates symmetry and |Z| > 1.96 indicates important asymmetry). The Sign test was used for significantly asymmetric data, with results presented as exact p-value and 95% confidence interval (CI) for the median difference. The Wilcoxon signed-rank test was used for symmetric data, with results presented as Z statistic, exact p-value, and effect size (r), where r = Z/√N and values of 0.1, 0.3, and 0.5 represent small, medium, and large effects, respectively. Both tests were performed using exact methods due to the small sample size.

Measure of lesional burden	Total affected area (cm²)	Number of lesions
Baseline median (IQR)	0.7 (0.5-2.8)	2 (1-4)
Final follow-up median (IQR)	0 (0-1)	0 (0-1)
Z-skewness of differences	-3.669	-1.902
Statistical test used	Sign test	Wilcoxon signed-rank test
Test statistic	Not applicable	Z = -3.496
Exact p-value	< 0.001	< 0.001
Effect size/95% CI	95% CI: 0.6306-2.7775	r = 0.903

The total affected area decreased noteworthy from a baseline median of 0.7 cm² (IQR: 0.5-2.8) to a final follow-up median of 0 cm² (IQR: 0-1). The significantly asymmetric distribution of differences (Z-skewness = -3.669) justified the use of the Sign test. The median reduction was 0.7 cm² (95% CI: 0.6306-2.7775), showing a statistically significant reduction (p < 0.001).

Similarly, the number of lesions showed a meaningful reduction from a baseline median of 2 (IQR: 1-4) to 0 (IQR: 0-1) at final follow-up. The symmetric distribution of differences (Z-skewness = -1.902) justified the use of the Wilcoxon signed-rank test, which showed statistical significance (Z = -3.496, p < 0.001) with a large effect size (r = 0.903).

Collectively, these results demonstrate a statistically substantial and a pivotal clinical improvement in both key objective measures of lesional burden following the intervention. It should be noted that these promising findings are derived from a preliminary cohort with a small sample size (N = 15) and therefore require validation in larger, prospective studies to confirm their generalizability.

## Discussion

This retrospective case series provides novel evidence on the use of intralesional bleomycin for periungual warts in a Nicaraguan population, a group underrepresented in the dermatological literature. A key strength of this study is the use of serial, quantitative measurement of lesion area (cm^2^) as the primary efficacy endpoint, an approach not previously applied specifically to periungual warts treated with bleomycin. This methodology enables a more sensitive and objective assessment of treatment response than the binary or categorical clearance scales commonly used in prior studies.

A comprehensive literature search was conducted on November 14, 2025, across PubMed/MEDLINE, DOAJ, SciELO, LILACS, and Google Scholar using predefined search strings that combined terms related to bleomycin and cutaneous warts with Boolean operators (AND, OR, NOT) to exclude genital warts, anogenital conditions, and review-type publications (e.g., systematic reviews, meta-analyses). This systematic electronic database search yielded 542 records. Subsequently, a supplementary snowball searching strategy was employed between November 18 and 23, 2025. This involved backward (reference list) searching of the initially identified relevant articles, which yielded two additional records, and forward (citation) searching using tools like Google Scholar's "cited by" function, which identified 11 more records. In total, 555 articles were retrieved for screening. Of these, 44 studies met the predefined inclusion criteria: reporting original data on periungual or other cutaneous warts treated with bleomycin in humans, published in English or Spanish, and excluding case reports [28]. A selection of representative studies is summarized (Table 4). Notably, none of the included studies employed serial quantitative lesion area measurement as a primary efficacy endpoint, underscoring the methodological novelty of the present study [14-27].

**Table 4:** Summary of selected studies reporting the use of intralesional bleomycin (IL) for the treatment of cutaneous warts: efficacy and assessment methods This table includes the study population, wart type and location, bleomycin regimen (concentration in mg/mL or U/mL, volume per wart, number of sessions), and efficacy outcomes, with specification of the measurement method used to assess treatment response: binary (complete vs. not complete), categorical (e.g., complete, partial, no response), or semiquantitative (ordinal or percentage-based). Outcomes include complete (total clearance), partial (partial clearance), and recurrence (reappearance of warts). Only representative articles were included due to space constraints.

Author (year)	Sample/warts	Wart type/location	Bleomycin regimen	Efficacy (measurement type)
Kumar et al. (2019) [23]	183 patients, 703 warts	Common: plantar 42.1%, dorsal 33.7%, palmar 14.6%, periungual 9.5%, mosaic 1.05%	1 mg/mL IL; 0.1-1 mL/wart; up to 2 injections at two-week intervals; ≤2 mg/session	Complete: 95.16% warts, 85.24% patients; partial: 3.4%; recurrence: 1.42%; categorical
Di Chiacchio et al. (2019) [15]	43 patients	Single-digit ungual: periungual 77.3%, subungual 22.7%	3 U/mL IL; 0.1 mL; single session; ± electroporation	Group A: 50%; Group B: 85.7% complete cure; binary
Puri (2020) [16]	20 patients, 50 warts	Resistant: palmoplantar 56%, periungual 44%	1 mg/mL IL + 2% lignocaine; 0.5-1 mL/wart; up to three sessions at two-week intervals	Complete: palmoplantar 71.4%, periungual 90.9%; binary/dichotomous
Singal and Grover (2020) [17]	80 patients, 250 warts	Ungual: periungual 74.8%, subungual 25.2%; mostly hands	3 U/mL IL; 0.1-0.3 mL; 3-4-week intervals	Complete 100%; recurrence 3.2%; binary
Shrikant et al. (2020) [20]	200 patients, 468 warts (bleomycin)	All cutaneous types: common, plantar, palmar, filiform, plana, periungual	1 mg/mL IL; max 4 sessions, 3-week intervals	Complete 77.99%, partial 10.47%, no response 11.53%; categorical
Kumar and Sharma (2021) [27]	42 patients, 118 warts	Plantar 40, palmar 31, periungual 11, common 35, mosaic 1	1 mg/mL IL; 0.1–0.2 mL/lesion; 2 sessions at 2–4 weeks	Complete 94.9%, partial 4.2%, no response 0.8%; categorical
B et al. (2021) [24]	90 patients, 305 lesions	Palmar 148, plantar 48, periungual 40, multiple 69	1 mg/mL IL; 0.2–1 mL; repeated after 2 weeks if residual; max 2 mL/session	Complete 82% lesions; overall cure 94.5%; binary
Marahatta et al. (2021) [19]	19 patients, 53 warts	Resistant periungual, palmar, plantar, palmoplantar	1 mg/mL IL; up to three monthly sessions; max 2 mL/session	Complete 89.5%, near-complete 10.5%; recurrence 15.8%; semiquantitative ordinal
Sachan et al. (2023) [21]	35 patients, 107 warts	Resistant palmo-plantar	1 mg/mL IL; 0.2-1 mL; up to four sessions every two weeks	Complete 90.6%, partial 5.6%, no response 3.7%; semiquantitative (% wart clearance)
Rahmatullah et al. (2023) [14]	154 adults	Cutaneous: palmoplantar and periungual	0.1% IL bleomycin (1 mg/mL) + 2% lignocaine; injected until blanching	Complete 94.8% at six weeks; binary
Singh et al. (2023) [25]	90 patients, ≥4 warts each	Verruca vulgaris, plana, palmoplantar, periungual, filiform, subungual	1 mg/mL IL; 0.2-1 mL/wart; up to three injections at two-week intervals	Complete 70%, partial 20%, no response 10%; categorical
Ranjan et al. (2024) [22]	51 patients, 146 warts	Resistant periungual and palmoplantar	1.5 mg/mL IL; 0.2-1 mL; 1-2 injections at two-week intervals	Complete 72.5%, near-complete 25.6%, recurrence 1.9%; categorical and percentage-based ordinal
Singh et al. (2025) [18]	54 patients, 52 completed	Resistant/difficult cutaneous: palmar/plantar 74.1%, periungual 25.9%	1 mg/mL IL + 2% lignocaine; 0.1-2 mL/wart; max two sessions at four-week intervals	Complete 94.2% at 12 weeks; partial 5.5%; no response 3.8%; categorical
Samal et al. (2025) [26]	56 patients, 54 warts	Periungual warts	1 U/mL IL + 2% lignocaine; 0.1-1 mL/wart; max 2 mL/session; follow-up 4, 12, 24 weeks	Complete 74.1% at 4 weeks, 97.3% at 12 weeks, 96.3% at 24 weeks; partial 22%, no response 3.7%; categorical via photographic comparison

In this cohort, 60% of patients achieved complete clinical remission, while all patients demonstrated either complete or partial improvement, resulting in a 100% overall response rate. These findings are consistent with the high efficacy reported for intralesional bleomycin in larger prospective and retrospective studies of periungual and palmoplantar warts, with complete clearance rates exceeding 85% in most reports [18,27]. Randomized controlled trial data further support the superiority of bleomycin over cryotherapy, with reported efficacy rates of 94.8% versus 74%, respectively [14]. High clearance rates have also been documented in studies focusing exclusively on ungual warts, including series reporting complete resolution after a limited number of treatment sessions [17]. Combination approaches may further enhance therapeutic outcomes, as evidenced by significantly higher cure rates when bleomycin is combined with electroporation compared with bleomycin monotherapy [15]. The absence of recurrence among complete responders during six months of follow-up in the present study suggests durable treatment effects and compares favorably with the low recurrence rates reported in the literature [18,19].

The principal methodological contribution of this study lies in its quantitative evaluation of treatment response. Measurement of total lesion area reduction allowed detection of clinically meaningful improvement in patients who did not achieve complete clearance. This is particularly relevant given evidence that larger baseline lesion size is associated with poorer therapeutic response [18,23]. The observed median lesion area reduction of 0.7 cm² provides a more granular and statistically robust assessment of treatment efficacy. Such quantitative metrics may be especially valuable in future studies evaluating combination therapies, optimizing dosing strategies, or conducting comparative effectiveness research against other intralesional agents, such as 5-fluorouracil [21].

The safety profile observed was consistent with existing evidence. Transient pain, erythema, and post-inflammatory hyperpigmentation were the most frequently reported adverse effects and resolved without intervention, in line with prior studies [14,16,19]. No cases of Raynaud phenomenon were observed, in contrast to isolated reports in the literature [16]. Nail unit complications, including onychomadesis and one case of permanent partial nail loss, underscore the anatomical vulnerability of the periungual region. These effects are likely related to cytotoxic exposure of the nail matrix and emphasize the importance of precise, low-volume intralesional injection techniques to minimize matrix involvement. Notably, studies employing meticulous injection methods have reported no permanent nail damage [18,23]. The observed negative correlation between treatment duration and percentage area reduction suggests that early therapeutic response may predict faster remission, whereas lesions requiring prolonged therapy may be inherently more resistant. This observation aligns with previous findings linking shorter disease duration and smaller lesion size to improved outcomes [16,18] .

Several limitations must be acknowledged. The small sample size and retrospective design limit generalizability and preclude causal inference, a limitation shared by other retrospective analyses in this field [19]. The absence of a control group prevents direct comparison with alternative therapies, although existing randomized data favor bleomycin over cryotherapy and suggest superior efficacy compared with other intralesional agents for palmoplantar warts [14,21]. Additionally, bleomycin was evaluated exclusively as monotherapy, and the potential additive benefit of combination approaches such as electrochemotherapy could not be assessed [15].

The quantitative methodology also has inherent constraints. Lesion area estimation based on two-dimensional measurements does not capture volumetric changes and may underestimate true disease burden in raised periungual warts. Future studies should incorporate three-dimensional assessment techniques, including high-frequency ultrasonography or digital reconstruction, to improve response quantification. Demographic homogeneity and contextual ethical limitations may further affect external validity.

In conclusion, this study supports intralesional bleomycin as an effective and generally well-tolerated treatment for recalcitrant periungual warts, consistent with robust international evidence [17,27]. The use of quantitative lesion area measurement provides a sensitive and reproducible outcome metric that may strengthen future clinical trials. Prospective randomized studies with larger and more diverse populations are warranted to validate these findings, compare treatment modalities, and optimize delivery techniques using quantitative response parameters [18].

## Conclusions

This study demonstrates intralesional bleomycin robust effectiveness and generally well-tolerated therapeutic intervention for recalcitrant periungual warts. The treatment achieved a significant rate of complete clinical remission and a universal overall response, accompanied by a predictable and predominantly transient adverse effect profile. The introduction of serial, quantitative lesion area measurement provides a sensitive, objective, and novel metric for assessing treatment response in this challenging anatomical location, addressing a substantial methodological gap in the existing literature. While the findings are constrained by the study's retrospective design and limited sample size, they contribute valuable real-world evidence from an underrepresented population and support the integration of intralesional bleomycin into the therapeutic modalities for resistant periungual warts. Future prospective and controlled studies utilizing such quantitative outcomes are warranted to further optimize treatment protocols and validate these results in broader, more diverse cohorts.
